# An Improved YOLOv8-Based Foreign Detection Algorithm for Transmission Lines

**DOI:** 10.3390/s24196468

**Published:** 2024-10-07

**Authors:** Pingting Duan, Xiao Liang

**Affiliations:** 1 School of Information Engineering, Minzu University of China, Beijing 100081, China; 2 Key Laboratory of Ethnic Language Intelligent Analysis and Security Governance of MOE, Minzu University of China, Beijing 100081, China

**Keywords:** power transmission lines, foreign object detection, YOLOv8, feature fusion

## Abstract

This research aims to overcome three major challenges in foreign object detection on power transmission lines: data scarcity, background noise, and high computational costs. In the improved YOLOv8 algorithm, the newly introduced lightweight GSCDown (Ghost Shuffle Channel Downsampling) module effectively captures subtle image features by combining 1 × 1 convolution and GSConv technology, thereby enhancing detection accuracy. CSPBlock (Cross-Stage Partial Block) fusion enhances the model’s accuracy and stability by strengthening feature expression and spatial perception while maintaining the algorithm’s lightweight nature and effectively mitigating the issue of vanishing gradients, making it suitable for efficient foreign object detection in complex power line environments. Additionally, PAM (pooling attention mechanism) effectively distinguishes between background and target without adding extra parameters, maintaining high accuracy even in the presence of background noise. Furthermore, AIGC (AI-generated content) technology is leveraged to produce high-quality images for training data augmentation, and lossless feature distillation ensures higher detection accuracy and reduces false positives. In conclusion, the improved architecture reduces the parameter count by 18% while improving the mAP@0.5 metric by a margin of 5.5 points when compared to YOLOv8n. Compared to state-of-the-art real-time object detection frameworks, our research demonstrates significant advantages in both model accuracy and parameter size.

## 1. Introduction

With the continuous growth in electricity demand, the construction of overhead transmission lines has rapidly expanded [1,2,3]. The power industry is a crucial part of the national economy, especially the stable operation of transmission lines, which is vital for the quality and safety of electricity transmission within the power grid [4,5]. The environment around transmission lines is complex, often affected by foreign objects such as bird nests, plastic trash, kites, and balloons. These objects can cause short circuits [6,7], leading to faults in the power system, and they pose economic and safety risks [8,9]. According to data from the State Grid of China, incidents caused by these foreign objects rank second only to lightning and external force incidents among all causes of power outages [10]. Therefore, the timely detection and handling of these foreign objects are crucial for ensuring the safety of transmission lines.

With the accelerated development of China’s power grid infrastructure, the construction of transmission lines has become increasingly complex and dense [11]. Traditional manual inspections, due to high operational risks and heavy workloads, can no longer meet current demands. In practical applications, manual inspections are greatly limited by safety risks and inefficiency. Although unmanned aerial vehicles (UAVs) and similar equipment have improved the safety and efficiency of inspections to some extent, the onboard detection algorithms lack real-time processing capabilities and unbiased training data, significantly hindering their adaptability to complex scenarios [12]. Therefore, there is an urgent need for more intelligent detection methods that efficiently integrate inspection and detection tasks to meet the practical needs of modern power grid construction [13].

In recent years, artificial intelligence (AI)-based technologies have been widely applied to foreign object detection tasks. However, these tasks face multiple challenges: on the one hand, the targets to be detected on transmission lines are irregular in size, with sparse and unevenly distributed samples; on the other hand, since transmission lines are typically installed across diverse geographical areas, the acquired images often have complex and variable backgrounds. Moreover, existing methods are hindered by high computational costs. To address these challenges, this paper proposes a GCP-YOLO algorithm (an improved of YOLOv8 incorporating GSCDown, CSPBlock, and PAM) for detecting foreign objects on transmission lines. The main contributions of this article are summarized as follows.

1. This study constructs the Transmission Line Foreign Object Detection (TL-FOD) dataset, which contains 2817 images, through techniques such as hue, saturation, and value (HSV) enhancement, random blur and noise addition, and simulated weather conditions. Additionally, utilizing the AIGC platform effectively addresses the issue of sample imbalance. This dataset provides comprehensive and authentic data support for foreign object detection on power transmission lines.
2. In this study, the algorithm improves with targeted modifications to address the challenges in transmission line detection. Specifically, the GSCDown module replaces standard convolution by utilizing dimensionality reduction and expansion techniques, which not only reduce parameters and computational costs but also integrate multi-scale features, thereby improving computational efficiency and addressing the challenge of varying target sizes. The CSPBlock, designed with cross-stage partial connections and grouped convolutions, captures richer semantic information while maintaining relatively stable computational complexity. To improve the recognition of different objects, the introduced PAM highlights key information without increasing parameters, effectively distinguishing between background and target, and ensuring high accuracy even in the presence of background noise. As a result, compared to the original model, the improved architecture achieves an 18% reduction in parameters and a 4% reduction in computational cost while improving detection accuracy (mAP@0.5) from 84.1% to 88.5%.
3. Employing a distillation strategy that integrates feature, response, and relational knowledge enhances detection accuracy, enabling the student network to extract more information from the teacher network, ultimately improving mAP@0.5 to 89.6%.

In foreign object detection for transmission lines, image sensors are the key components for obtaining high-quality image data. The effectiveness of the proposed improved YOLOv8 algorithm relies on high-quality datasets, which are sourced from high-resolution image sensors. Commonly used sensors include CMOS (complementary metal-oxide semiconductor) and CCD (charge-coupled device) sensors, which are capable of capturing foreign object information in the complex environments surrounding transmission lines at high resolutions and frame rates. These sensors are typically mounted on drones or ground-based monitoring equipment, allowing for 24/7 image acquisition around the transmission lines, thereby providing stable and reliable data support for detection tasks.

The paper is structured as follows. Section 2 discusses the current literature regarding object detection. Section 3 describes the proposed GCP-YOLO network. Section 4 provides detailed information about the proposed TL-FOD dataset and analyzes the experimental results. Finally, Section 5 concludes the work.

## 2. Related Work

Currently, image-based foreign object detection methods for transmission lines can be categorized into three main types: methods based on traditional hand-crafted features, methods based on machine learning, and methods based on deep learning.

### 2.1. Traditional Foreign Object Detection Methods

Researchers employ conventional algorithms for the detection of foreign object intrusions in power transmission lines. C. Chen et al. utilize adaptive filtering techniques to eliminate terrain interference and employ Euclidean clustering algorithms to segment the detection results, thereby constructing a recognition model for foreign object intrusions in power lines [14]. L. Cheng et al. introduce an algorithm based on distance estimation focused on accurately calculating the coordinates of foreign objects [15]. Concurrently, S. Jiao et al. integrate the Euclidean distance method with a predictive region drift strategy to enhance the accuracy of foreign object detection [16]. Despite these advancements, traditional algorithms have limitations in recognizing types of foreign objects, noise resistance, and the diversity of target segmentation, increasing the complexity of detection.

### 2.2. Classical Machine Learning-Based Detection Methods

Consequently, numerous scholars explore the use of machine learning methods such as multilayer perceptrons (MLPs) and support vector machines (SVMs) for the detection of foreign objects. S. Z. Wu et al. propose a cascaded structure based on MLP [17], while F. Mahdi Elsiddig Haroun et al. enhance the feature set of SVMs using satellite imagery to improve detection efficiency [18]. X. Ye et al. employ a particle swarm optimization-enhanced SVM for detection purposes [19]. Although machine learning models require extensive feature engineering, their data mining capabilities may be inferior to those of deep learning models. However, the superior high-level feature extraction and end-to-end solutions provided by deep learning present new possibilities for the detection of foreign objects in power transmission lines.

### 2.3. Deep Learning-Based Foreign Object Detection Methods

With the rapid development of high-performance computing and artificial intelligence, intelligent and automated detection of foreign objects on transmission lines has become an important research area. These methods can automatically learn features and identify the location of foreign objects. Depending on the stage of the process, these methods can be classified into multi-stage detectors and end-to-end detectors.

**Multi-Stage Detectors.** Liang et al. develop a method for detecting foreign objects in power transmission lines based on the Faster R-CNN framework [20]. Guo et al. [21] first constructed a dataset of transmission line images and then employed a region-based convolutional neural network (Faster R-CNN) to detect foreign objects such as fallen items, kites, and balloons. This method can handle foreign objects of various shapes, demonstrating excellent generalization ability. Despite the high accuracy of the two-level networks, the processing speed is slow and not suitable for real-time detection.

**End-to-End Detectors.** In contrast, single-stage networks such as SSD and the YOLO series demonstrate higher detection speeds and efficiency. Li et al. replace the backbone network of YOLOv3 with Mobilenetv2 to reduce the number of parameters [22], albeit at the expense of some detection rate. Song et al. enhance performance by integrating k-means clustering and DIoU NMS with YOLOv4 [23,24]. Huang et al. utilize YOLOv5s in conjunction with Ghost convolution and KL divergence loss [25,26]. Liu et al. incorporate attention mechanisms and ASPP modules into YOLOX to improve detection accuracy [27]. Meanwhile, Yu et al. combine hyperparameter optimization and SPD convolution with YOLOv7 to enhance the accuracy of detecting small targets [28], although this results in slower detection speeds. Yang et al. [29] developed a foreign object detection algorithm based on a denoising convolutional neural network (DnCNN) and YOLOv8. They consider various types of foreign objects, such as bird nests, kites, and balloons, achieving a mean average precision (mAP) of 82.9%. However, there remains significant room for improvement in detection performance, and the diversity of categories in the dataset requires further enhancement.

Both end-to-end and multi-stage algorithms have achieved favorable results on the given dataset. Multi-stage detection algorithms exhibit a clear advantage in terms of detection accuracy, although they also require considerable computational resources and processing time. In contrast, end-to-end detectors are trained using illumination models, aiming to strike a balance between detection accuracy and speed. Our work falls under the category of end-to-end detectors.

Given the constraints on computational resources and the need for rapid processing in practical deployments, the choice is made to utilize YOLOv8n [30]. This model balances minimal parameter and computational demands with high accuracy and speed. Deep learning approaches rely heavily on large quantities of high-quality data. However, the challenges of class imbalance and data scarcity in the detection of foreign objects in power transmission lines limit both model training and accuracy. Consequently, research shifts towards refining algorithms and enhancing data. Algorithm refinement includes techniques such as zero-shot learning and transfer learning [31,32], while data augmentation methods encompass image enhancement, GAN-based techniques [33], and generative model approaches [34].

## 3. Proposed Method

**Figure 1:**
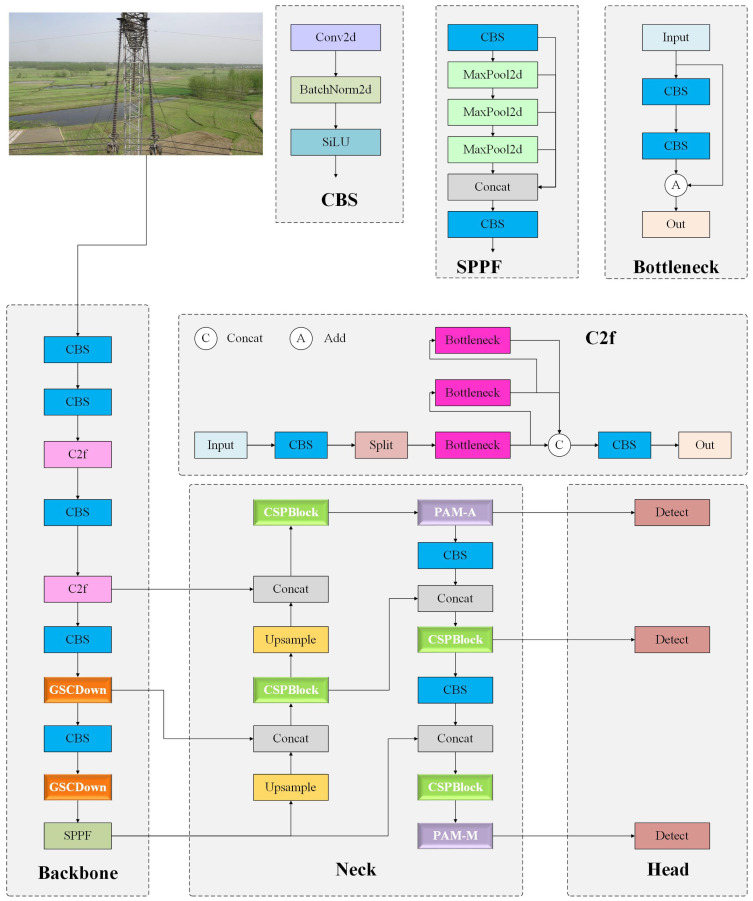
The network structure of GCP-YOLO, an improved YOLOv8 algorithm incorporating GSCDown, CSPBlock, and PAM.

**Figure 2:**
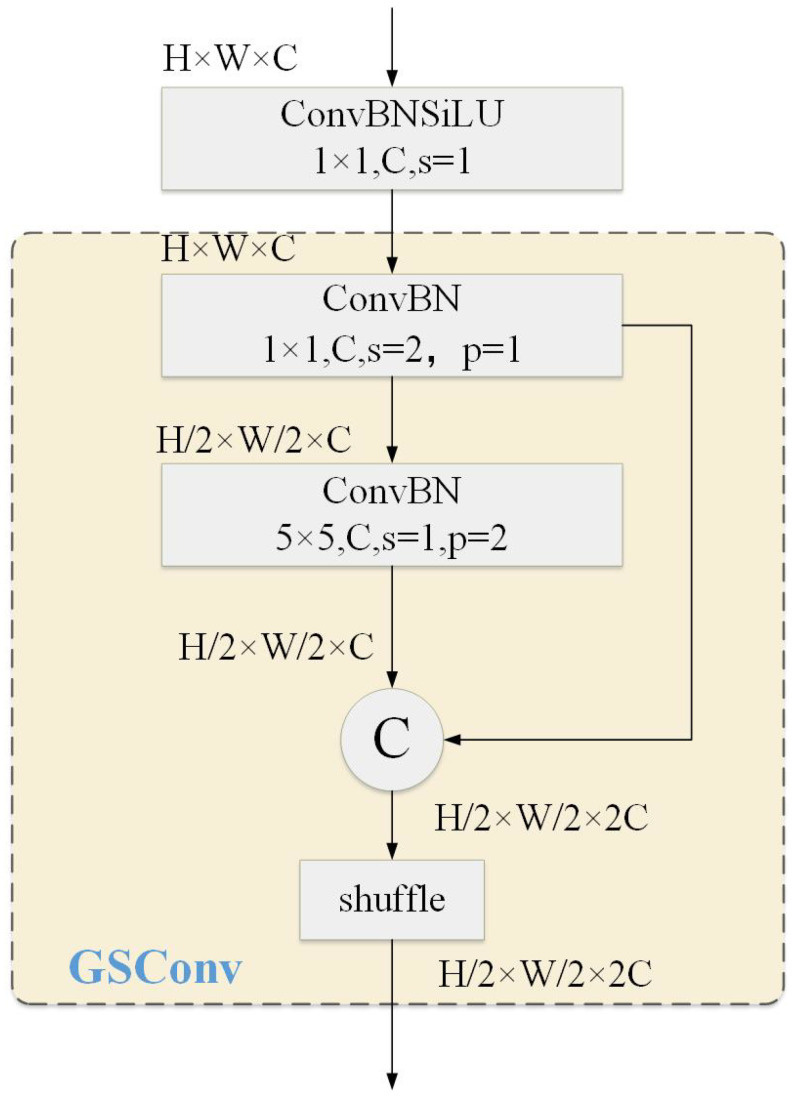
The structure of GSCDown.

**Figure 3:**
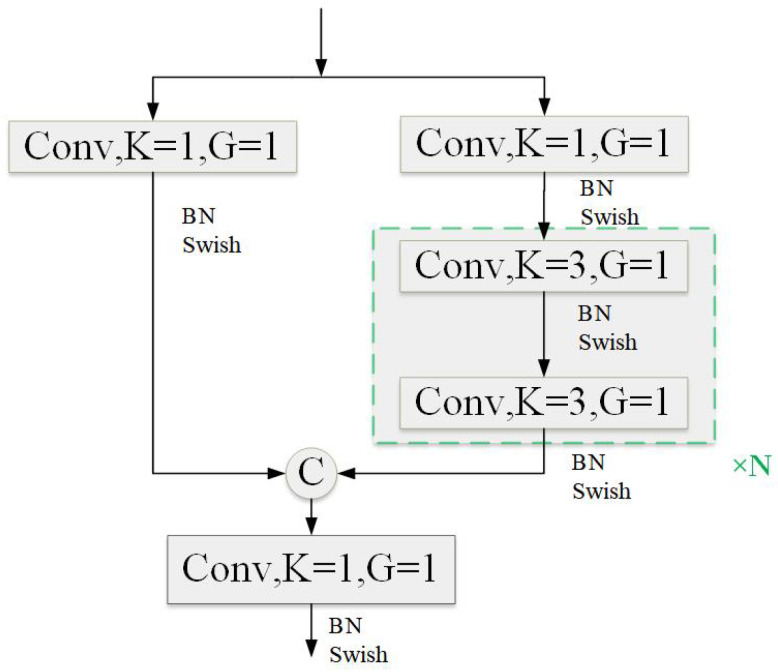
The structure of CSPBlock.

**Figure 4:**
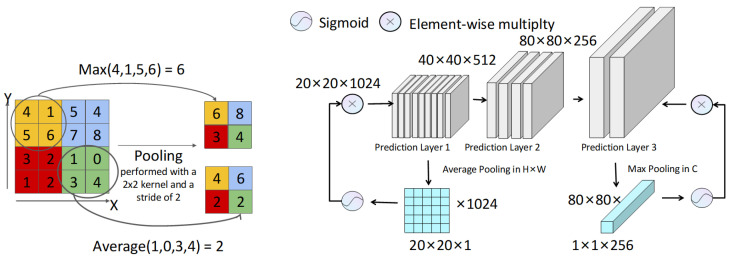
The feature map of dimensions 20×20×1024 from prediction layer 1 is condensed to 20×20×1 via the average pooling, while the 80×80×256 feature map from prediction layer 3 is compressed to 1×1×256 through the max pooling. Subsequently, the resultant feature map undergoes depth and size expansion under the constraints of the sigmoid function, which is employed for element-wise multiplication with the original feature map.

The improved network framework, GCP-YOLO, is illustrated in Figure 1. Specifically, in the feature extraction module, we introduced the GSCDown module (as shown in Figure 2), which decouples spatial reduction and channel augmentation operations, significantly reducing both the number of parameters and computational complexity. This approach not only enhances detection efficiency but also improves detection accuracy for foreign objects. In the feature fusion module, we utilize the CSPBlock to replace the C2f structure in YOLOv8 (as shown in Figure 3). The cross-stage connections are employed to ensure the transmission and fusion of semantic information between feature maps at different scales, thereby enhancing feature representation. To supervise the recognition of different targets with varying receptive fields, we incorporate PAM-M and PAM-A modules in front of the small-scale and large-scale detection heads, respectively (as shown in Figure 4). These modules emphasize key information without introducing additional parameters, allowing the model to accurately distinguish between background entities and target entities, which is particularly advantageous for addressing complex background scenarios commonly found in transmission line images. Finally, a knowledge distillation strategy is employed using the YOLOv8x as the teacher model to further improve the model’s accuracy.

### 3.1. Ghost Shuffle Channel Downsampling

Detecting foreign objects on power transmission lines presents significant challenges due to the diversity and complexity of these objects. Foreign objects can vary greatly in shape, size, and material, including bird nests, ice layers, plastic bags, kites, and balloons. The variations of these objects under different environmental conditions further complicate detection. Additionally, the widespread distribution of transmission lines necessitates a detection system that can rapidly process large volumes of data with limited computational resources to achieve real-time and efficient detection.

YOLOv8 employs 3×3 standard convolutions for spatial downsampling and channel transformation, but this approach significantly increases computational cost and the number of parameters. Specifically, the computational cost of YOLOv8 is $O(9HWC^{2})$, and the number of parameters is $O(18C^{2})$, where *H*, *W*, and *C* represent the height, width, and number of channels of the feature map, respectively. To address this issue, we introduce the GSCDown module, which enhances downsampling efficiency by separating spatial reduction and channel expansion operations. As shown in Figure 2, the GSCDown module first uses a 1×1 convolution layer to halve the number of input feature map channels, reducing computational complexity and the number of model parameters, thereby improving system computational efficiency. It then employs the GSConv [35] structure to perform grouped convolutions, lowering computational complexity while maintaining its feature representation ability. GSCDown integrates global context pooling with standard convolutional downsampling, reducing feature map resolution (and computational complexity) while ensuring that critical contextual information is retained when the model processes global features. This helps in recognizing complex foreign objects, reducing computational cost and preserving downsampling information. Consequently, it maintains high performance while reducing latency.

The design of the GSCDown module reduces the number of parameters in YOLOv8 from $O(18C^{2})$ to $O(10C^{2}+25C)$, significantly decreasing the parameter count and ensuring efficient system operation in resource-constrained environments, thereby meeting the demands of real-time detection. The GSCDown module is particularly suited for the challenging application of detecting foreign objects on power transmission lines. Through innovative convolution design, it enhances the object recognition ability while maintaining high precision, significantly improving system computational efficiency and achieving more efficient downsampling.

### 3.2. Cross-Stage Partial Block

Feature Pyramid Network is a key technology in the field of object detection, enhancing detection performance by integrating features at different resolutions [36,37,38]. The conventional FPN [36] introduces a top-down pathway to fuse multi-scale features. However, considering the limitations of unidirectional information flow, PAFPN [38] adds a bottom-up path aggregation network, which improves feature fusion effectiveness.

In the process of model fusion for foreign object detection on power transmission lines, the C2f structure, which primarily relies on 3 × 3 convolutional layers and a simple feature segmentation and fusion method, is inadequate for effectively capturing and representing small and complex foreign object features. Although the C2f structure demonstrates good computational efficiency, this efficiency is achieved at the cost of reduced feature representation abilities. Additionally, C2f performs feature segmentation and fusion at a single scale, lacking the ability to fuse multi-scale features, which is crucial for detecting foreign objects of varying sizes and shapes—a capability that C2f lacks. In contrast, the CSPBlock (Cross-Stage Partial Block), derived from CSPNet (Cross-Stage Partial Network) [39], enhances feature fusion through an innovative design involving cross-stage partial connections and grouped convolutions, thereby improving feature representation while maintaining relatively stable computational costs. The CSPBlock structure is depicted in Figure 3. Specifically, CSPBlock divides features into two parts: one part is directly transmitted through cross-stage connections, while the other part undergoes complex convolutional operations before being fused. This design not only reduces computational burden but also preserves rich feature information. Grouped convolutions reduce the number of parameters and computational load, while dense connections ensure efficient information transmission.

In the context of foreign object detection on power transmission lines, the introduction of CSPBlock significantly enhances the model’s feature representation abilities. By employing a cross-stage partial connection mechanism, CSPBlock ensures efficient transmission and fusion of features across different stages, thereby improving detection accuracy and robustness. Furthermore, CSPBlock’s spatial awareness is markedly improved. The use of grouped convolutions and dense connections maintains the integrity and continuity of spatial information during feature fusion, enabling the model to perform better in handling complex backgrounds and multi-scale features. This is particularly beneficial in the complex environments of power transmission lines, where it effectively distinguishes between background and foreign objects. Additionally, the design of CSPBlock effectively mitigates the vanishing gradient problem, ensuring efficient gradient transmission in deep networks, which enhances the stability and convergence speed of model training. Finally, CSPBlock strikes a balance between model lightweightness and real-time performance. It enhances feature representation capabilities without significantly increasing computational load, making it suitable for scenarios requiring long-term monitoring and efficient foreign object detection on power transmission lines.

### 3.3. Pooling Attention Mechanism

Power transmission line images often face complex background clutter challenges, including elements such as sky and vegetation that are irrelevant to the subject, as well as large structures like towers and wires that may serve as backgrounds for small targets like bird nests. In such visual environments, accurately identifying and separating key target objects places higher demands on the neural network’s representation capabilities. However, increasing network depth typically accompanies a significant rise in parameter count, conflicting with the goal of model lightweighting. Therefore, this section introduces PAM to enhance the model’s accuracy without increasing computational burden, thereby better addressing the complex background issues in images of foreign objects on power transmission lines. The pooling attention mechanism is shown in Figure 4.

Pooling layers, as parameter-free fixed operations, can maintain certain feature invariance. Specifically, average pooling helps reduce the increased estimation variance due to limited neighborhood size, while max pooling mitigates the estimation bias caused by parameter errors in convolutional layers [40]. During the decoding prediction process, the network outputs feature maps of sizes 80×80×256, 40×40×512, and 20×20×1024, respectively. If the receptive field is too small, excessive local information retrieval may lead to the loss of large targets. Conversely, if the receptive field is too large, small targets may be overlooked. Therefore, appropriately designing the size of the receptive field is crucial to ensuring accurate target detection.

Channel attention mechanisms compress spatial dimensions of feature maps while retaining channel dimensions, focusing on solving object recognition problems. Conversely, spatial attention mechanisms compress channel dimensions while preserving spatial dimensions of feature maps, focusing on solving object localization problems. In shallower networks, due to limited receptive fields, the network may overly emphasize local information, making small targets easily captured by the network and leading to misidentification of some large targets as background. To improve this, this study introduces a channel attention mechanism that enhances the representation of local details by focusing on information interaction between different channels. It utilizes average pooling to extract more global features from local information, thereby better distinguishing foreground from background.

As the number of layers in a neural network increases, the receptive field covered by the feature maps gradually enlarges, potentially leading to the neglect of smaller targets by the network. To address this issue, a spatial attention mechanism is introduced to enhance the detection ability for small targets. In this section, max pooling is employed to simulate the spatial attention mechanism. Max pooling can retain key features and extract finer-textured information, thereby effectively capturing small targets and reducing missed detections.

### 3.4. Feature Distillation

Knowledge distillation is a model compression technique that allows a smaller student model to learn from and achieve improved performance by mimicking a pre-trained, larger teacher model, as highlighted by Hinton et al. [41]. In the knowledge distillation process, the teacher model’s outputs (typically the softmax layer’s output, i.e., the probability distribution) serve as the target for training the student model, a process referred to as “distillation”. Through this method, the student model can learn the decision-making process of the teacher model, even without direct access to the original training data. Knowledge distillation can be based on different types of knowledge, including response-based knowledge (i.e., the teacher model’s predicted outputs), feature-based knowledge (activations from the intermediate layers of the teacher model), and relation-based knowledge (the relationships between different layers or samples).

**Figure 5:**
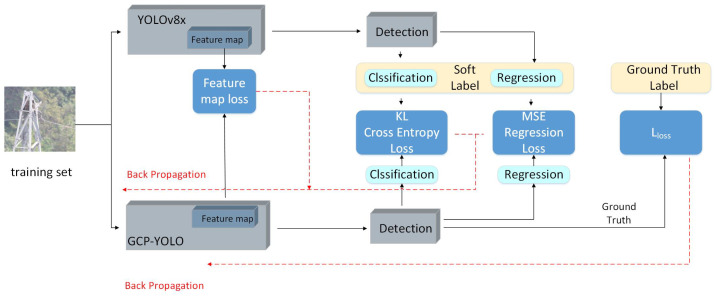
Schematic diagram of knowledge distillation.

The GCP-YOLO network is trained using a knowledge distillation algorithm, as depicted in Figure 5. The transmission line training set is simultaneously input into both the GCP-YOLO network and the YOLOv8x model for forward propagation. During this process, both the teacher and student networks obtain corresponding feature maps from the feature extraction layers. To ensure that the lightweight student model, GCP-YOLO, extracts feature information equivalent to that of the teacher network, a feature map loss function is constructed using the feature maps of both networks. Through back-propagation, the feature loss is gradually minimized, thereby enhancing the feature extraction ability of the lightweight network. Similarly, the Kullback–Leibler (KL) divergence loss function and mean square error function are utilized to construct a prediction probability loss function and regression result loss function between the teacher and student networks. This guides the student network to learn from the teacher network, allowing it to match the teacher network’s classification and regression performance. As seen in the structure diagram, the recognition results of the lightweight GCP-YOLO model exhibit classification loss, regression loss, and confidence loss, collectively represented by loss *L*. Through back-propagation, the gap between the recognition results of the lightweight GCP-YOLO network and the ground truth is minimized. Since the teacher network, YOLOv8x, is pre-trained, the back-propagation process only occurs within the student network, i.e., the GCP-YOLO network.

We conducted a quick validation experiment to select the most suitable distillation method for our GCP-YOLO model. The results are shown in Table 1. Our conclusion is that CWD (content and weighted dissimilarity) is better suited for our model, while MGD underperforms compared to mimicking due to its complex hyperparameters, which reduce its generalizability. During the feature distillation process, the loss ratio is set to 1.0 to fully leverage the feature distillation loss in optimizing the student model. Experimental results show a significant improvement in model performance through feature distillation, with the mean average precision (mAP) increasing by 1.5%. These substantial improvements confirm the effectiveness of feature distillation in the YOLO algorithm and present a new approach for optimizing model performance. Notably, the use of a constant decay factor combined with CWD’s feature loss type provides us with an efficient feature distillation strategy.

## 4. Results and Analysis

### 4.1. Dataset Preparation

Using high-precision localization and intelligent analysis techniques, this research meticulously designs and constructs a dataset for detecting foreign objects on power transmission lines. The composition of the TL-FOD dataset is presented in Table 2. The dataset encompasses four common types of foreign objects: bird nests, kites, balloons, and plastic bags. Additionally, the insulator category is included to enhance the algorithm’s recognition abilities, totaling 1401 images. To augment the dataset’s diversity and robustness, HSV enhancement techniques are employed to simulate various lighting conditions in the images. In practical applications, object detection systems may encounter extreme weather conditions such as rain, snow, and fog. Incorporating these factors during model training can enhance the system’s real-world performance. To simulate raindrop shapes and dynamics, we applied noise generation and motion blur, adjusting raindrop density and length to represent varying intensities of rainfall. Snowfall is simulated using random noise and Gaussian blur to mimic the distribution and depth of snowflakes, with varying degrees of blur to depict snowflakes at different distances. Fog is simulated by adjusting transparency and contrast using an exponential decay formula, gradually blurring distant objects and reducing overall contrast to reflect diminished visibility.

To address the issue of sample imbalance, we employed Photoshop’s generative fill feature, utilizing Adobe’s AI-generated content (AIGC) technology to effectively augment the dataset. AIGC leverages artificial intelligence to automatically generate various forms of content, including text, images, audio, and video. Photoshop’s generative fill, an intelligent image editing tool developed by Adobe based on AIGC, allows users to select specific areas of an image and automatically generate content that seamlessly blends with the surrounding environment using AI algorithms. This feature enables image restoration, expansion, or object removal. Adobe has enhanced this function by integrating generative adversarial networks (GANs) and deep learning models, making the generative fill more intelligent and natural, thereby simplifying complex image editing workflows. The specific operational steps are as follows.

**Figure 6:**
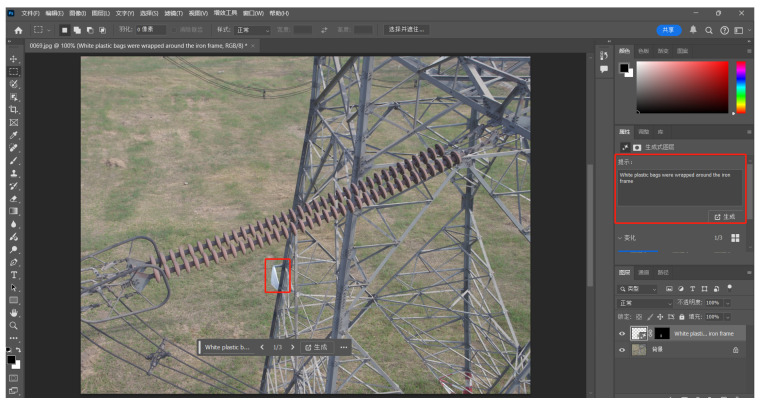
The Photoshop “Generate” image process.

1. **Area Selection:** Using Photoshop’s selection tools (such as the rectangular marquee or lasso tool), the user marks the area of the image where content needs to be modified or generated.
2. **Text Description Input:** After selecting the area, the user can input a natural language description of the desired content in the provided text box. For example, inputting “White plastic bags were wrapped around the iron frame” allows the AI to analyze both the text description and the context of the selected area, generating content that meets the user’s expectations.
3. **AI-Generated Image:** Upon confirming the text input, the user clicks the “Generate” button, and Photoshop produces the final image (as shown in Figure 6).

Additionally, to create class labels, we utilized a visual image annotation tool called “labelImg”. The annotation results are saved in XML format according to the PASCAL VOC standard, covering five categories: bird nests, kites, balloons, plastic bags, and insulators.

**Figure 7:**
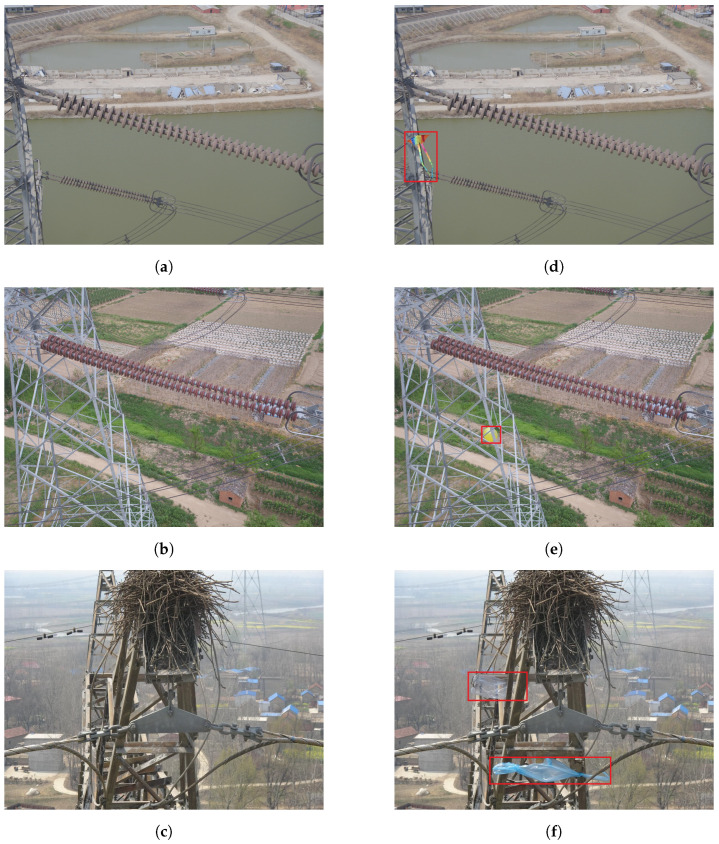
Examples of objects generated by AIGC: (**a**–**c**) are the original images, and (**d**–**f**) are the generated kite, balloon, and plastic bag based on (**a**–**c**), respectively.

Figure 7 illustrates the data generated using AIGC technology. Through meticulous construction and augmentation, the TL-FOD dataset has been successfully created. It is an image collection focused on foreign object detection on power transmission lines. This dataset comprises a total of 2817 images, including 1401 original images and 1426 augmented images. It has been systematically divided into a training set of 2502 images, a validation set of 157 images, and a test set of 158 images. This division ensures the efficiency of model training and provides a solid foundation for the initial evaluation of model performance and the testing of its generalization ability.

### 4.2. Experimental Environment and Hyperparameter Settings

The experiments in this study are conducted using the PyTorch deep learning framework. In terms of hardware configuration, the GPU used is an RTX 3090, and the CPU is a 13th Gen Intel^®^ Core™ i9-13900HX. The experimental setup is detailed in Table 3.

### 4.3. Evaluation Metrics

To comprehensively evaluate the detection performance of the proposed improved model, a range of evaluation metrics have been employed. These metrics encompass accuracy, recall rate, mAP@0.5, mAP@0.95, model parameter count, model size, and detection speed. Some of the evaluation metrics utilize the following parameters in their formulas: TP (true positives, predicted as positive samples and are indeed positive), FP (false positives, predicted as positive but are actually negative), and FN (false negatives, predicted as negative but are actually positive).

1. Precision: Precision measures the proportion of correctly predicted positive samples to the total number of predicted positive samples, indicating the precision of the model.
  (1) $$P=\frac{TP}{TP+FP}$$
2. Recall: Also known as the true positive rate, recall denotes the proportion of actual positive samples that are correctly predicted as positive by the model, reflecting the model’s ability to recognize positive samples.
  (2) $$R=\frac{TP}{TP+FN}$$
3. mAP: Mean average precision is the average of precision values across different recall levels. It combines information from both precision and recall to measure the model’s performance. mAP@0.5 represents the mAP value at an IoU threshold of 0.5, while mAP@0.95 represents the average mAP over IoU thresholds ranging from 0.5 to 0.95 (usually with a step size of 0.05). mAP is a comprehensive metric for evaluating the performance of detection algorithms. In this study, it refers to the average detection accuracy of all categories of transmission line anomalies. A higher mAP value indicates better overall performance of the algorithm.
  (3) $$mAP=\frac{1}{n}\sum_{i=1}^{n}AP_{i}$$
4. FPS: Frames per second is used in object detection to measure the speed at which a system processes images, specifically referring to the number of image frames processed per second. In practical applications, the processing time per frame may include image preprocessing time, model inference time, and post-processing time, among others. The calculation formula for FPS is based on the time taken to process each frame.
  (4) $$FPS=\frac{1}{t_{\mathrm{per}\;\mathrm{frame}}}$$

### 4.4. Experimental Results

#### 4.4.1. Ablation Experiment

To comprehensively evaluate the impact of different components on model performance and to validate the effectiveness of the improvement strategies adopted in this study, detailed ablation experiments are conducted. Ablation experiments systematically remove or add model components to determine the contribution of each component to the final model performance.

The detection results of the ablation study model are shown in Table 4. The focus of the experiments is to examine the impact of three technical modules—GSCDown, CSPBlock, and PAM—on model performance. By ablating one or more of these modules, different models are constructed, and their performance in detection tasks is observed, specifically reflected in key metrics such as mAP@0.5, mAP@0.95, model size, parameter count, and GFLOPs. The experimental results indicate that the inclusion of different components significantly affects model performance. For instance, when only the GSCDown module is introduced, the model’s mAP@0.5 improves from the baseline of 84.1 to 87.2, while the parameter count is reduced by 0.5 M, demonstrating that this module not only enhances detection accuracy but also optimizes the model’s parameter efficiency. Similarly, adding the PAM module also notably improves both mAP@0.5 and mAP@0.95 scores. When combining multiple modules, particularly using GSCDown, CSPBlock, and PAM together, the model achieves the highest performance with 88.5 mAP@0.5 and 66.9 mAP@0.95 while maintaining a small model size and low computational complexity.

#### 4.4.2. Distillation Experiment

In this study, distillation experiments are conducted to evaluate the impact of various temperature coefficients (t) on model performance. The results, as summarized in Table 5, clearly demonstrate the positive effect of the distillation process on mean average precision (mAP@0.5). Specifically, the model exhibits optimal performance when the temperature coefficient is set to 2, achieving a 4.1% increase in mAP@0.5.

Table 5 details the experimental outcomes at different temperature coefficients, utilizing contrastive weighted (CWD) feature loss with a fixed loss ratio of 1.0. The table indicates that, as the temperature coefficient decreases, the mAP@0.5 value of the model first increases and then decreases. At a temperature coefficient of 1, the model reaches the highest mAP@0.5 value of 89.6%, representing a significant enhancement compared to the baseline model without distillation (88.5%).

These findings underscore the importance of hyperparameter selection during model fine-tuning and provide valuable insights for future research. The optimal performance at a temperature coefficient of 2 validates the efficacy of this coefficient in the current experimental configuration. Subsequent studies can further explore the effects of different distillation strategies and hyperparameter settings on model performance, aiming to achieve higher accuracy and generalization abilities.

#### 4.4.3. Comparative Experiment

The comparative experimental results in Table 6 demonstrate that the proposed network architecture outperforms other existing YOLO object detectors in the task of foreign object detection on power lines. A detailed comparison of the performance between the YOLOv8n model and the proposed method is conducted, as shown in Table 7. The proposed method, compared to the YOLOv8n model, achieves an improvement of 3 to 5 percentage points in mAP across various detection categories. Particularly in the task of detecting foreign objects on power transmission lines, precision increases to 95.5% for the plastic bag category, significantly improving detection efficiency. This enhancement provides a robust technical foundation for deployment in real-world application scenarios.

### 4.5. PAM

**Figure 8:**
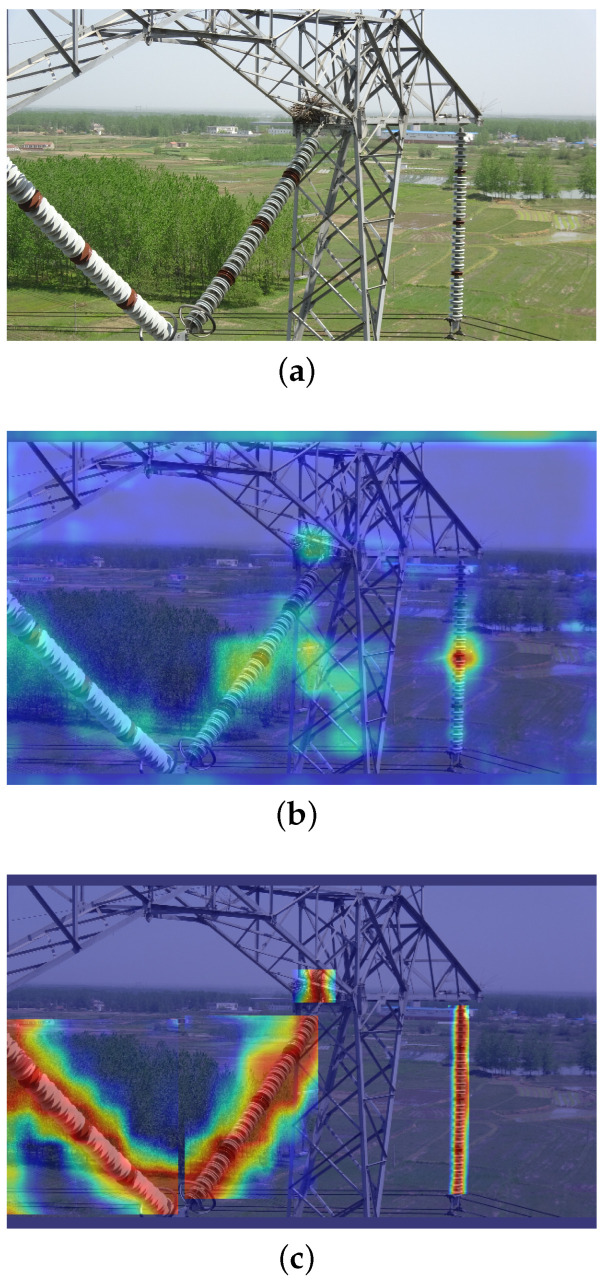
(**a**) Original image; (**b**) heat map of YOLOv8n; (**c**) heat map of our model.

GradCAM is an effective visualization tool used to generate heatmaps [45]. Through the reverse propagation mechanism of GradCAM, the model’s output class confidence can be converted into gradient values, visualizing the gradient intensity of feature maps in the heatmap. Deeper red shadows indicate areas the model focuses on more, while deeper blue shadows indicate areas of lower attention. As depicted in Figure 8, the experimental outcomes distinctly reveal the disparities in feature attention among various object detection models. Observations from the results indicate that the YOLOv8n model demonstrates a deficiency in allocating attention to diminutive objects and exhibits a relatively low sensitivity to objects situated at a distance. In stark contrast, the model introduced in this study has shown exceptional performance in mitigating background noise, with a pronounced concentration of attention on the central regions of objects. This refined attention allocation mechanism significantly enhances the accuracy of the model in bounding box prediction tasks, thereby substantially improving the overall detection performance. Furthermore, this improvement not only bolsters the model’s robustness but also provides a robust technical foundation for performance optimization in practical applications.

## 5. Discussion

Compared to the original YOLOv8 model, the enhanced GCP-YOLO model significantly improves the accuracy of foreign object detection in power transmission lines. However, some foreign objects remain undetected, potentially due to the following factors.

Firstly, environmental complexity is a key factor. Power transmission lines located in outdoor environments are susceptible to weather conditions such as fog, rain, snow, and variations in lighting, which may hinder the detection efficiency of the model. Secondly, the diversity in size and shape of foreign objects poses a challenge, particularly when the sample data are limited. Smaller or irregularly shaped objects may be difficult for the model to identify, as their features may not be salient enough. Thirdly, the limitation of the dataset is an issue. The training dataset includes only common types of foreign objects, leading to the model’s inability to recognize rare or unknown objects.

In this study, we construct a Transmission Line Foreign Object Detection (TL-FOD) dataset, which encompasses images captured under varying resolutions and weather conditions. The richness of the dataset is largely attributed to advancements in sensor technology, particularly the application of high-resolution CMOS sensors. During model training, we simulate different lighting conditions, blur, and noise environments to ensure the model’s robustness in real-world applications. This approach effectively addresses common image quality issues encountered by sensors when capturing images in outdoor settings.

In future work, we will explore how more advanced sensor technologies can further enhance the accuracy of detection algorithms. For instance, integrating infrared sensors or multispectral imaging technology could improve the detection of foreign objects under extreme environmental conditions. Moreover, employing higher-resolution sensors and more intelligent image processing techniques will further enhance the practical application of the YOLOv8 algorithm in transmission line inspections.

## Figures and Tables

**Table 1:** Studies of the distillation methods for GCP-YOLO on the TL-FOD validation dataset. The baseline mAP@0.5 of the student model is 88.5.

Methods	Epochs	mAP@0.5
Mimicking [42]	300	87.6
MGD [43]	300	84.3
CWD [44]	300	89.6

**Table 2:** The composition illustration of the TL-FOD dataset.

Dataset	Number
Original images	1401
HSV enhancement	470
Scene augmentation (rain, snow, fog)	470
AIGC-generated images	476
TL-FOD (total images)	2817

**Table 3:** Key parameters set during the model training process.

Parameters	Setup
Epochs	300
Momentum	0.937
Learning rate	0.01
Weight decay	0.0005
Batch size	32
Wiou	False
Input image size	640 × 640
Optimizer	SGD
Data enhancement	Mosaic

**Table 4:** Results of the ablation experiment, where a ‘✓’ indicates that the corresponding technique is used to construct the model for detection.

GSCDown	CSPBlock	PAM	mAP@0.5	mAP@0.95	Model Size	Params	GFLOPs
			84.1	64.1	6.3 MB	3.2 M	8.1
✓			87.2	67.5	5.6 MB	2.7 M	7.7
	✓		84.3	64.5	6.3 MB	3.0 M	8.1
		✓	86.5	64.9	6.3 MB	3.2 M	8.1
✓	✓		87.4	64.9	5.6 MB	2.7 M	7.7
	✓	✓	86.6	66.1	6.3 MB	3.0 M	8.1
✓		✓	85.2	66.1	5.6 MB	2.7 M	7.7
✓	✓	✓	88.5	66.9	5.6 MB	2.7 M	7.7

**Table 5:** Effect of distillation on mAP@0.5.

t	Feature Loss Type	Feature Loss Ratio	Before Distillation	After Distillation
10	CWD	1.0	88.5	85.5
5	CWD	1.0	88.5	87.1
2	CWD	1.0	88.5	87.9
1	CWD	1.0	88.5	89.6

**Table 6:** Comparison of detection performance of various models.

Model	mAP@0.5	mAP@0.5:0.95	Precision	Recall	Params	FLOPs	FPS
YOLOv5-n	82.6	61.3	89.3	73.7	1.9 M	4.5 G	64
YOLOv5-s	82.9	62.5	77.5	80.5	6.6 M	15.8 G	56
YOLOv5-m	84.8	67.7	85.9	74.9	19.9 M	47.9 G	50
YOLOv6-n	84	64.4	77.7	78.1	4.2 M	11.8 G	102
YOLOv6-s	84.6	67.8	85.1	74.7	17.2 M	44.0 G	86
YOLOv6-m	85.4	68.5	79.1	77.5	52.0 M	161.2 G	40
YOLOv7tiny	81.3	60.4	80.8	73.3	6.0 M	13.1 G	120
YOLOv7	84.5	67.8	88.6	75.4	36.0 M	103.2 G	36
YOLOv8-n	84.1	64.1	88.6	75.6	3.2 M	8.1 G	176
YOLOv8-s	86.2	69.4	84.2	79.1	10.6 M	28.4 G	85
YOLOv8-m	88.2	70.8	89.9	77.0	24.6 M	78.7 G	51
YOLOv9-t	86.4	65.9	89.7	76.5	2.6 M	10.7 G	120
YOLOv9-s	86.7	66.8	85	75.9	9.6 M	38.7 G	73
YOLOv9-m	89.4	71.1	80.3	78.3	32.5 M	130.7 G	34
YOLOv10	84.6	63.6	87.9	75.9	2.7 M	8.2 G	178
YOLOv10-s	87.1	66.9	90.1	80.1	8.0 M	24.5 G	151
YOLOv10-m	87.6	69.8	87.3	80.7	16.4 M	63.4 G	125
**Ours**	**89.6**	**67.7**	**83.4**	**81.2**	**3.6 M**	**12.0 G**	**142**

**Table 7:** Comparison of mAP for different categories.

Category	YOLOv8n		Ours	
	mAP@0.5 (%)	mAP@0.5:0.95 (%)	mAP@0.5 (%)	mAP@0.5:0.95 (%)
All	84.1	64.3	89.6	69.5
Bird nest	89.6	52.0	92.4	63.2
Balloon	78.2	56.7	85.0	54.4
Kite	77.9	60.1	82.5	67.4
Plastic bag	84.3	78.0	95.5	85.4
Insulator	90.5	73.7	92.6	77.2

## Data Availability

The TL-FOD dataset can be downloaded from: http://gofile.me/7c7UU/SOug1AxyI (accessed on 12 September 2024).
